# Cancer Doctors

**Published:** 1875-07

**Authors:** 


Cancer Doctors have some "ways that are dark and tricks that are vain," like Bret Harte's "Heathen Chinee." Their system appears to be the same in outline wherever the rascals exist, and is essentially as follows:

1. He who aspires to the dignity of a "Cancer Doctor" must provide himself with an efficient caustic or caustics, which may be used in the form of a powder, paste or plaster.

2. The quack must disguise the articles by suitable coloring materials, and stoutly deny that they are caustics.

3. He must advertise largely that the great Dr. Tumorsmash will "take out the cancers without the use of knife or caustic, and without pain," and guarantee the cure.

2. When the patients flock in, he must pronounce everything a cancer, whether it be a wart, corn, fatty tumor, ulcer, necrosis, sarcoma, or what not.

Now he is ready, and his success will depend upon his enterprise in carrying out the plan.

Honest John Leatherhead, the shoemaker, has observed a lump upon the surface of his thigh, in fact an encysted tumor. It gets in the way of his lapstone, gets hit with his pegging hammer, and, when it is hammered too much, becomes swollen and tender. Mrs. Leatherhead has read Dr. Tumorsmash's handbill, and advises John to go and see him. John, who has eyed the swelling for many a month with fear and suspicion, determines to have a talk with the great man, especially as his "consultations are free." He dresses in his best, and presents himself. The quack views him from head to foot, and draws him out into conversation, to gauge the "size of his pile." He then looks at the encysted tumor, and, with solemn and ponderous dignity, pronounces it a cancer of the most malignant type. John turns pale, and his leather head shakes on his shoulders. Can he be cured? Yes, says Dr. Tumorsmash, but it will cost $500. Leatherhead pleads poverty, but finally pays $100 in advance for a guaranteed cure.

"I believe you take out cancers without pain," says he.

"Certainly," replies the quack, "that I always do. However, I must put you under a course of preliminary treatment for a a few days. This preliminary treatment is a little painful, but you can bear it, and then I will remove the tumor without the slightest suffering."

Dr. Tumorsmash now administers a good dose of morphine; next he surrounds the tumor with a circle of adhesive plaster to protect the skin, and claps on his caustic. This hurts severely, but then it is only "preliminary treatment," and meanwhile he plies the morphine vigorously to keep Leatherhead as quiet as possible. After a proper number of hours, when he judges that he has produced a sufficiently deep slough, he removes the caustic and applies poultices. The pain now ceases, and the poultices are continued until the eschar is fully separated from the living flesh. Now the quack is ready for his grand operation of removing the tumor "without pain." The patient's friends assemble, the poultice is removed, and the surface sponged clean. Dr. Tumorsmash discourses as follows: "My friends I have called you in to see one of the triumphs of modern science. If you look at this tumor, you will see how wonderfully my medicine discriminates between the cancer and the the natural flesh. Look at this deep grove (pointing to the line of demarkation.) That was the boundary of the cancer, and you see that this wonderful remedy has followed the disease everywhere exactly up to that line, and has nowhere gone a hair beyond it. It has the property of killing the cancer to its remotest roots, and has no effect on healthy flesh. I will now proceed to remove it." Suiting the action to the word, he gracefully seizes the rotten mass with his forceps, and lifts it out of its cavity "without pain."

Leatherhead is amazed and profoundly grateful. In the course of three months the ulcer is healed; and for the rest of his life he and Mrs. Leatherhead never weary of sounding the praises of Dr. Tumorsmash.

